# Lipedema associated with Skin Hypoperfusion and Ulceration: Soft Tissue Debulking Improving Skin Perfusion

**DOI:** 10.1055/a-2181-8469

**Published:** 2024-03-04

**Authors:** Feras Alshomer, Seok Joon Lee, Yeongsong Kim, Dae Won Hong, Changsik John Pak, Hyunsuk Peter Suh, Joon Pio Hong

**Affiliations:** 1 Department of Plastic and Reconstructive Surgery, University of Ulsan, College of Medicine, Seoul Asan Medical Center, Seoul, Republic of Korea

**Keywords:** lipedema, hypoperfusion, liposuction, ulcer

## Abstract

Lipedema is a progressive connective tissue disease with enlargement of adipose tissue, fibrosis, fluid collection, and dermal thickening. Herein, we present a case of lipedema associated with skin hypoperfusion and ulceration in which soft tissue debulking with liposuction improved patients' symptoms. A 39-year-old female presented with asymmetric progressive initially unilateral lower limb swelling with severe pain with subsequent skin ulceration. Conservative management failed to improve her condition. After excluding other causes and detailed radiologic investigation, lipedema was diagnosed with an associated impaired skin perfusion. Trial of local wound care and compression therapy failed to improve the condition. Subsequent soft tissue debulking with circumferential liposuction and ulcer debridement and immediate compression showed dramatic improvement of the symptoms and skin perfusion. The unique nature of this case sheds light on lipedema as a loose connective tissue disease. Inflammation and microangiopathies explain the associated pain with hypoperfusion and ulceration being quite atypical and in part might be related to the large buildups of matrix proteins and sodium contents leading to fragility in microvessels with frequent petechiae and hematoma and subsequent tissue ischemia. Conservative measures like compression therapy plays a significant role in disease course. Surgical debulking with liposuction was shown to be efficacious in reducing the soft tissue load with improvement in limb pain, edema, circumference, and skin perfusion that was seen in our patient. Lipedema is a frequently misdiagnosed condition with disabling features. Skin involvement in lipedema with potential hypoperfusion was shown and it requires further investigation.

## Introduction

Lipedema is a poorly recognized loose connective tissue disease with an estimated prevalence of approximately 11 to 18.8% of population, with females being primarily involved. 1 2 The disease is characterized by diffuse enlargement of subcutaneous adipose tissue together with fibrosis and increase in extracellular fluid collection. 1 The subcutaneous fatty deposition primely involves the lower extremities and extends from the buttock to the ankles with rare involvement of the feet. 3 The diagnosis of lipedema is quite challenging as high index of suspicion is warranted and is based on certain clinical criteria that typically describe bilaterally symmetric limb pain and heaviness with easy bruising and enlargement together with minimal edema. 4 The cutaneous manifestations of lipedema were shown to involve skin hypothermia together with telangiectasia without known association with ischemia or ulceration, which was related to the microangiopathic nature the disease is associated with. 5 The condition was shown to have a strong association with hormonal imbalances, for instance, following pregnancy or menopause and usually begins around puberty explaining the high prevalence in females. 2 Men can be affected as well; majority of reported cases involved patients with altered hormonal profile alongside high estrogen and low testosterone levels as in cases of liver disease or hypogonadism. 6 7 Lipedema can be confused with different conditions that primarily involve lower extremity swelling like lymphedema or in relation to cardiac, renal, or hepatic disease as well as venous insufficiency or obesity. For that, meticulous workup and approach is warranted for accurate diagnosis. 8

Herein, we describe an atypical presentation of lipedema in a patient who presented with an asymmetric initially unilateral progressive circumferential lower limb swelling with pain and heaviness and associated skin ulceration and hypoperfusion that has failed multiple attempts of local wound care with lack of clear diagnosis despite detailed workups in other institutes for which subcutaneous tissue debulking eventually improved the condition. The obscure clinical scenario and management is further discussed.

## Case

A 39-year-old female presented initially for further evaluation of a unilateral left lower limb pain, swelling, and heaviness with an associated recurrent skin ulceration that started 4 years prior to her presentation. During the assessment and management of the left lower limb, the condition progressed to involve the contralateral right lower limb with heaviness, pain, and swelling. The patient history revealed that she has been otherwise healthy apart from a previous history of left unilateral lower extremity deep venous thrombosis for which she underwent a 6-month course of anticoagulation therapy 10 years prior to her presentation. Subsequently, she was doing fine until the gradual onset of progressive left lower limb swelling, pain, and heaviness that was aggravated by walking and later progressed to develop an associated skin ulceration. The patient indicated her concerns with the condition affecting her life, in which multiple visits to several health care institutes failed to reach a proper management.

**Fig. 1:**
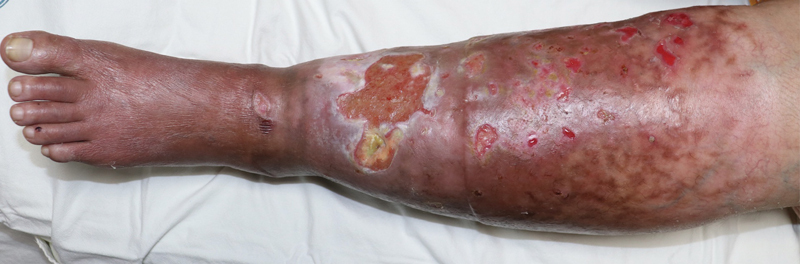
Initial clinical presentation of the patient. Frontal view of the patient's left leg s howing scattered areas of skin ulceration located mostly on the anterior and medial sides of the leg with poorly granulating base. The largest ulcer is located on the distal anterior third of leg measuring approximately 10 × 6 cm with its base showing deep extension to subcutaneous fat.

**Fig. 2:**
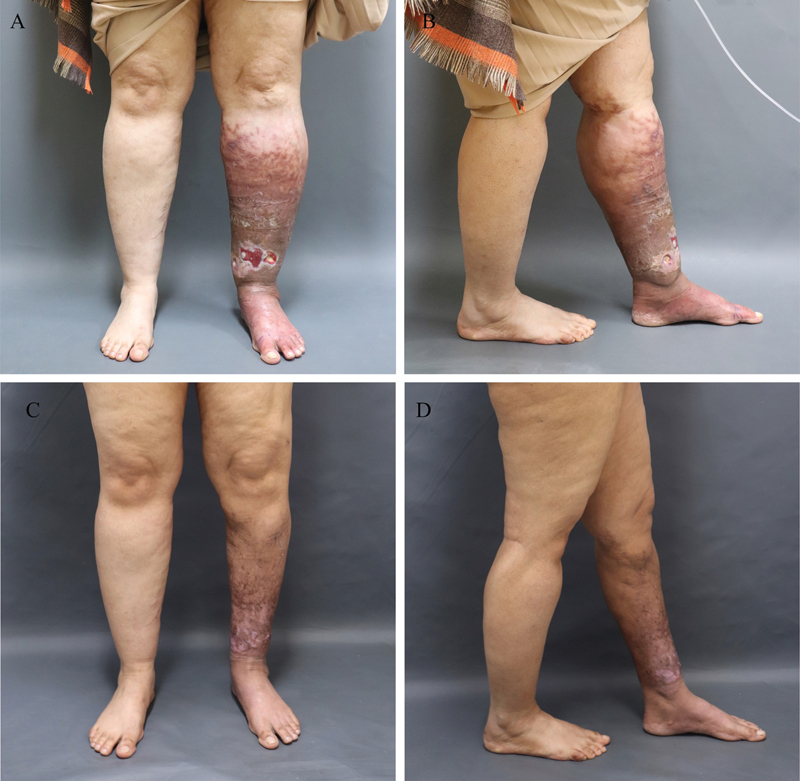
Clinical assessment of the patient. Clinical photos on preoperative assessment of the patient with frontal view ( **A** ) and lateral view ( **B** ) are shown following a trial of local wound care and hyperbaric oxygen therapy prior to surgical debulking with liposuction. Slight improvement of lower limb skin ulceration of all ulcers, except the largest, is shown. Follow-up clinical assessment photographs 8 months postoperatively are shown in ( **C,** **D** ) with frontal and lateral views, respectively. Clear improvement in limb condition with complete ulcer healing and reduction in limb width is also shown.

**Fig. 3:**
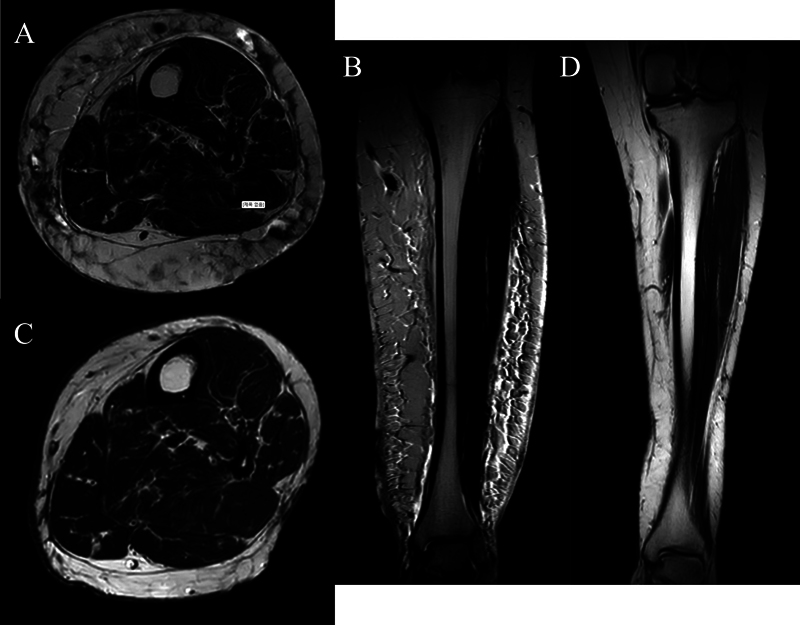
Magnetic resonance imaging assessment. Preoperative imaging workup with axial ( **A** ) and sagittal ( **B** ) views showing circumferential fatty hypertrophy with thickened hyperintense dermis and extensive reticular edema in T2 views. Follow-up assessment postoperatively showed decreased subcutaneous fat and fluid contents in both axial ( **C** ) and sagittal ( **D** ) T2 views.

**Fig. 4:**
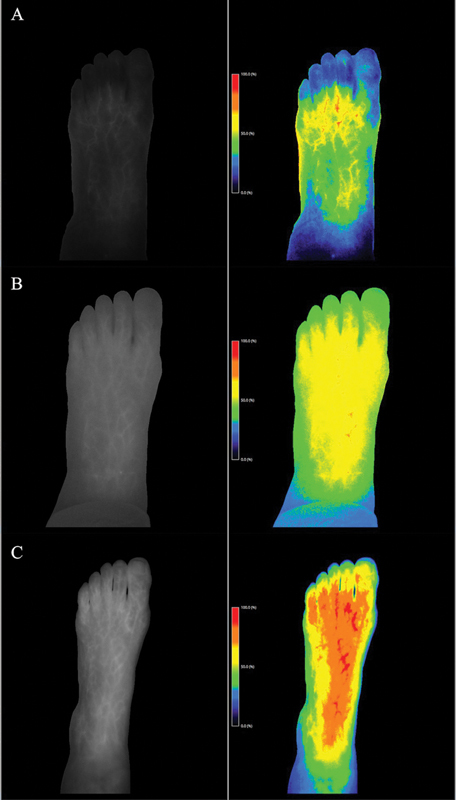
Indocyanine green skin perfusion assessment. Results of skin perfusion assessment preoperatively are shown in ( **A** ) with low signal intensity indicating impaired perfusion. Repeated assessment on day 2 postoperatively showed improved skin perfusion with better signal intensity as shown in ( **B** ). Follow-up assessment 8 months postoperatively is shown in ( **C** ) where significant improvement in skin perfusion is seen.

Physical examination showed that the patient has a body mass index of 27.2 with massively enlarged left leg swelling with coldness on palpation and minimal pitting edema together with circumferential erythematous skin changes. In association, there were multiple areas of skin ulceration scattered mostly on the anterior and medial sides of the leg with the largest located just on the distal anterior third of leg measuring approximately 10 × 6 cm. The ulcer bases were granulating with the largest ulcer having deep extension to subcutaneous fat with minimal discharge as shown in Fig. 1 . Circumferential measurements of the affected limb below the knee level with 5-cm increments were 47, 47, 42.5, and 38 cm with a calculated volume of 1.857 L. This was compared with the contralateral normal limbs which were 41.5, 41.5, 37.5, and 34 cm with a calculated volume of 1.452 L ( Fig. 2A, B ). Radiologic investigations showed patent arterial flow in both extremities on computed tomography angiography and no reflux in the superficial and deep veinous systems with no residual thrombosis. Lymphoscintigraphic evaluation of both lower extremities showed intact lymphatic system which ruled out lymphedema as a potential diagnosis. Magnetic resonance imaging (MRI) assessment showed diffuse circumferential fatty hypertrophy and thickened hyperintense dermis with extensive reticular edema in the left lower limb as shown in Fig. 3 . Perfusion assessment of the involved limb was done through indocyanine green (ICG) imaging and showed impaired foot tissue perfusion with low fluorescence signal intensity as shown in Fig. 4A . Informed consent was obtained from the patient for the participation and publications of manuscript-related materials.

The diagnosis of lipedema was suspected with impaired lower limb skin perfusion based on the detailed clinical and radiological assessment. The patient was initially managed with local wound care for the scattered ulcers in the lower limb with multiple sessions of hyperbaric oxygen therapy with partial improvement. This was only evident with epithelialization of all the ulcers, except the largest ulcer on the distal leg which exhibited enhanced granulation tissue with improved surrounding skin condition as shown in Fig. 2A, B . Compression therapy was started but the patient could not tolerate due to the associated pain. The patient was then booked for left leg soft tissue debulking with circumferential liposuction aiming to decrease subcutaneous adipose tissue and matrix load. The procedure was delivered through the use of ultrasound-assisted liposuction with tumescent fluid infiltration. Meticulous incision planning away from previously ulcerated and infected skin together with targeted fat aspiration from the deep fatty layer especially around previous ulcer sites was carried out with an aim not to spread any soft tissue infection. The procedure yielded a total of 2.25 L of fat aspirated from the affected limb followed by ulcer wound debridement and primary excision and closure together with immediate compression therapy postoperatively. On her follow-up, the patient tolerated the procedure remarkably with uneventful postoperative course. The limb condition has improved significantly and the subsequent limb circumferential measurements 8 months postoperatively were 34.5, 33, 31, and 26.5 cm with and estimated limb volume of 952 L as shown in Fig. 2C, D . Follow-up investigations showed reduction of subcutaneous fat and fluid content during MRI as shown in Fig. 3C, D . Repeated ICG skin perfusion assessment showed enhancement of skin perfusion immediately postoperatively with more dramatic improvement on her 8-month follow-up ( Fig. 4B, C ). The patient noticed significant improvement in her quality of life and with no ulceration recurrence as shown in Fig. 2C, D .

## Discussion

The aforementioned scenario is quite unique and sheds light on lipedema as a loose connective tissue disease. The distinctive nature of this case presenting with progressive asymmetric initially unilateral lower extremity swelling with severe pain, limb coldness and skin hypoperfusion, and ulceration is quite atypical and was the reason for the delayed management and the confusion to reach a proper diagnosis. The usual presentation of lipedema consists of bilateral simultaneous progressive disproportionate collection of connective tissue within the extremities with pain and tenderness initially that might advance to nodular formation and in extreme cases secondary lymphedema with other lymphedema-related features like cellulitis and papillomatosis and that was not the scenario in the presented case. 9

It is not clear if lipedema itself can predispose to skin hypoperfusion and ulceration; however, skin of lipedema patients is usually soft with hypertrophy of underlying dermis and lack of any epidermal changes with dryness and slow wound healing. 5 9 10 Only one case of lower extremity lipidemia was reported to have an associated skin ulceration, which was not directly related to lipidemia but rather to the patients' impaired limb sensation due to spina bifida with trauma as the cause of the ulcer. 11 To further assess the potential association between lipedema and skin hypoperfusion and ulceration, an assessment of lipedema-related connective tissue showed progressive buildups of glycosaminoglycans (GAGs) and sodium—both of which were linked to increased micro-blood vessels formation with defective hyperpermeable and dysfunctional phenotype leading to the overall microangiopathic condition. 1 This was significantly evident in earlier stages of disease before further progression into the development of an associated lymphedema at the last stage. 12 The high-sodium content was also evident during MRI and linked to the impairment of the barrier ability of endothelium with direct inflammatory changes and disruption of glycocalyx that lines vessels' wall. 13 14 15 16 Such vessel fragility might explain the frequent petechiae and hematoma formation with eventual tissue destruction and potential hypoxia lipedema patients are experiencing. 17 Moreover, similar blood pooling with accumulation of GAGs and sodium contents in the extracellular matrix was shown as a potential cause of tissue ulceration with an associated decrease in osmotic pressure and subsequent tissue hypoxia and ischemia, a mechanism that is also evident in venous ulcer formation. The association of venous insufficiency with lipedema is known with variable degree. 18 All these factors might explain the associated skin hypoperfusion and later ulceration and delayed wound healing that our patient came across. Moreover, this unique presentation is considered an extreme form of lipedema or with the potential possibility of secondary blood pooling and later venous congestion that might explain the disease process this patient is experiencing, thereby warranting a meticulous workup and approach. In our patient, we have shown on skin ICG perfusion assessment a decreased skin perfusion that was associated with diffuse fatty hypertrophy and fluids accumulation in subcutaneous tissue on MRI in which later improvement was evident on long-term follow-up as shown in Fig. 4 . After debulking, the excessive soft tissue load with significant reduction of the extracellular matrix content through circumferential liposuction and immediate compression to prevent further fluid re-accumulation was carried out. This was not amenable preoperatively because of the associated pain and tenderness that prevented the application of compressive therapy. Such pain was also explained by the microangiopathic condition with decreased blood supply to peripheral nerves and associated sympathetic nerve fibers inflammation. 19 Moreover, the efficacy of liposuction on decreasing the fatty tissue load and the associated improvement of venous pressure is not well investigated in previous literature and it is worth investigation in future work.

The distinctive presentation of lipedema, in general, with vague symptoms warrants high index of suspicion after excluding other causes of limb swelling like obesity, lymphedema, and chronic venous insufficiency. Management options of lipedema are limited and it classically resides on lifestyle modifications with weight reduction and early compression therapy. 20 21 22 For patients with disease progression or for those who failed conservative therapy, surgical intervention with lipectomy or liposuction might be considered. 23 The utility of liposuction was shown to be associated with reduction in associated limb pain, edema, pressure sensation, bruising, hematoma formation, and limb circumference with improved mobility and cosmesis as seen in our patient, though it is noncurative. 24 25 26 Moreover, meticulous surgical planning is warranted together with the use of microcannulas, vibrating cannulas, and tumescent local anesthesia not to cause any additional morbidities like secondary lymphatic injury. 27 28 In this patient, incision placement away from infected tissue as well as controlled plane of suction deep from ulcer base was made to decrease any chances of spreading of infection which led to an uneventful course postoperatively.

Lipedema is a frequently misdiagnosed condition with disabling features. The vague presentation of lipidemia mimicking other disease presentation warrants high index of suspicion with increased awareness about its prevalence among patients and health care providers. In this report, we showed a patient who suffered from an atypical form of lipedema with an asymmetric disease onset and skin hypoperfusion with secondary ulcerations where subsequent soft tissue debulking improved skin perfusion and patient symptoms. The unique involvement of connective tissue in the disease process in lipedema together with the associated microangiopathic nature and subsequent hypoxia and potential skin hypoperfusion with progressive fluid and tissue buildup require meticulous investigations in a study on a larger scale with long-term follow-up.
